# Draft Genome Sequence of the Iridescent Marine Bacterium *Cellulophaga lytica* CECT 8139

**DOI:** 10.1128/genomeA.00811-17

**Published:** 2017-09-07

**Authors:** Maylis Chapelais-Baron, Isabelle Goubet, Eric Duchaud, Eric Rosenfeld

**Affiliations:** a UMR 7266, CNRS, Littoral Environnement et Sociétés (LIENSs), Microbial Physiology Group, Université de La Rochelle, La Rochelle, France; b INRA, Virologie et Immunologie Moléculaires UR892, Université Paris-Saclay, Jouy-en-Josas, France

## Abstract

Some species of the genus *Cellulophaga* have been reported as having biotechnological interests and noteworthy physiological properties. We report here the draft genome sequence of *Cellulophaga lytica* CECT 8139, a bacterium that produces an intensely iridescent colony biofilm on agar surfaces.

## GENOME ANNOUNCEMENT

The genus *Cellulophaga* is a member of the family *Flavobacteriaceae* in the phylum *Bacteroidetes*. *Cellulophaga* strains have been isolated from a variety of coastline biotopes, including the water column, sediments, seaweed or diatom surfaces, and marine invertebrates (1–3). Some reports have focused on potential biotechnological properties of *Cellulophaga* species, including antifouling and algicidal activities (4–6), inhibition of quorum sensing (7), or the production of novel enzymes involved in the bioconversion of algal polysaccharides (e.g., the breakdown of carrageenans or agar) (8, 9). Other studies described the development of genetic tools for understanding the gliding motility mechanisms or for identifying the components involved in the type IX secretion system (T9SS) (10, 11).

*Cellulophaga lytica* is a yellow/orange-pigmented aerobic agarolytic carrageenolytic rod and is motile by gliding (12). *C. lytica* CECT 8139 (CL8139) was isolated from a red sea anemone on the Charente Maritime Atlantic coast (France) (13). On agar, strain CL8139 produces a colony biofilm that displays an intensely “pointillistic” iridescence under epi-illumination. Under conditions that mimic its natural environment, the cell population shows a unique periodicity that induces this structural coloration (14–16). However, the cellular and molecular mechanisms of this self-organization, as well as its biological role, are still unknown.

The draft genome sequence of *C. lytica* CL8139 was obtained using HiSeq 2000 (Illumina) sequencing (GenoScreen, France), which generated 129,354,414 (2 × 100 bp) paired-end reads assembled *de novo* in 87 scaffolds (>1 kb) using Velvet. The total size for the combined scaffolds is 3,773,420 bp, with an average G+C content of 31.97%. Gene prediction and functional annotation were carried out using the MicroScope annotation platform (https://www.genoscope.cns.fr/agc/microscope/home/index.php) (17). The *C. lytica* CL8139 draft genome is predicted to contain 3,321 protein-coding genes, a coding density of 91.11%, and an average gene length of 1,031 bp. Strain CL8139 is the third *C. lytica* strain whose genome is sequenced (12, 18). Using average nucleotide identity (ANI) (19) as a proxy index for genomic relatedness, strain CL8139 displays 99.22% and 99.21% identities with the *C. lytica* type strain LIM21 and with strain HI1, respectively, suggesting a very close intraspecies phylogenetic proximity. The core genome of the species *C. lytica* is composed of 2,951 protein-coding genes (>80% identity over >80% of the size of the smallest protein), and strain CL8139 encompasses 232 strain-specific genes (7.0%) that essentially encode proteins of unknown function, Rhs and Vgr family proteins, and their remnants, transposases, or restriction-modification systems.

Genome analysis identified a clustered regularly interspaced short palindromic repeat (CRISPR)-encoding locus, a *dnd* gene cluster likely acquired by horizontal genetic transfer (20), genes encoding the gliding motility machinery and the T9SS, as well as proteins similar to the cold-adapted, thermotolerant, and denaturant-stable GH5 endoglucanase Celal_2753 of *Cellulophaga algicola* IC166^T^ (21) and to the kappa-carrageenase CgkA of *Zobellia galactanivorans* (22).

In contrast to other marine *Flavobacteria* species, strain CL8139 is devoid of (i) key quorum-sensing genes (i.e., *luxS* and *luxI*), (ii) gene encoding the acyl-l-homoserine lactonase (AHL) recently identified in *Tenacibaculum* species (23), and (iii) proteorhodopsin-encoding genes (24).

The genome of this environmental bacterium may help identify the genes required for the production of iridescent colony biofilms, improve knowledge on genome evolution, and facilitate future functional genomics studies.

### Accession number(s).

This whole-genome shotgun project has been deposited in ENA under the accession number FZLT00000000. The version reported in this work is the first version, FZLT01000000.
